# Waugh Syndrome Presenting in Late Childhood: A Case of Atypical Age and Anatomy

**DOI:** 10.7759/cureus.105028

**Published:** 2026-03-11

**Authors:** Yenifer Almeida Tápanes, Javier Mora, Claudia A Garcia Gonzalez, Ana B Cuni Hernandez, Raysa Garces Ruiz, Jorge A Nunez Garcia

**Affiliations:** 1 Pediatric Surgery, Durazno Medical Assistance Center, Private Medical Assistance Institution for Professionals (IAMPP), Durazno, URY; 2 Surgery, Dr. Juan Guiteras Gener Medical University of Matanzas, Matanzas, CUB; 3 Critical Care, Faustino Pérez Hospital, Matanzas, CUB; 4 Family Medicine, Atlas Urology, Bradenton, USA; 5 Family Medicine, University of Medical Sciences of Camagüey, Camagüey, CUB

**Keywords:** intestinal malrotation, intussusception, ladd procedure, midgut volvulus, waugh syndrome

## Abstract

Waugh syndrome is the rare coexistence of intussusception and intestinal malrotation. We describe the case of a nine-year-old boy who initially presented with nonspecific gastrointestinal symptoms mimicking viral gastroenteritis. His condition worsened over several days, progressing to bowel obstruction. Imaging with contrast-enhanced CT confirmed an extensive ileocolic intussusception, although an associated malrotation was not recognized on the initial report. Given clinical deterioration, an exploratory laparotomy was performed. Intraoperative findings revealed a large ileocecal intussusception (involving the terminal ileum, appendix, cecum, and ascending colon) extending into the transverse colon, along with a midgut volvulus and underlying malrotation of the intestine. The intussusception was successfully reduced manually, an inflamed appendix within the intussuscepted segment was removed, and a Ladd’s procedure was carried out to correct the malrotation. The child recovered uneventfully and remained asymptomatic on follow-up. This case highlights an atypical age presentation of intussusception due to malrotation (Waugh syndrome) and underscores the importance of maintaining a high index of suspicion in older children. Early surgical intervention was critical in confirming the dual pathology and achieving a favorable outcome.

## Introduction

Waugh syndrome is a rare clinical entity defined by the coexistence of intestinal intussusception and congenital intestinal malrotation. It was first described by George Waugh in 1911, who noted the anatomical association between these two conditions long before modern imaging could confirm it. Despite more than a century since its initial description, the syndrome remains uncommon, with only a limited number of cases reported in the literature. Recent reviews suggest that fewer than 100 cases have been documented worldwide, underscoring its rarity and the diagnostic uncertainty it often creates, particularly in older children who fall outside the typical age range for intussusception [1]. 

Although classically described in infants, contemporary case reports demonstrate that Waugh syndrome can present across a broader age spectrum, including older children and adults. These atypical presentations frequently mimic benign gastrointestinal illnesses, leading to delays in diagnosis and treatment. Imaging may reveal subtle or initially overlooked signs of malrotation, such as a duodenum that fails to cross the midline or an abnormally positioned cecum. This highlights the importance of maintaining clinical suspicion when symptoms evolve from presumed viral gastroenteritis to progressive obstruction. Viral infections, particularly adenovirus and rotavirus, have been implicated as triggers for intussusception due to mesenteric lymphoid hyperplasia acting as a lead point. In children with unrecognized malrotation, this inflammatory cascade can precipitate a more complex clinical scenario, including volvulus or extensive ileocecal involvement. Recent pediatric studies have identified adenovirus as a significant risk factor for recurrent or complicated intussusception, reinforcing the interplay between infection and underlying anatomic vulnerability [2,3]. 

Timely surgical intervention remains essential for preventing ischemia and ensuring favorable outcomes in Waugh syndrome. Standard management includes reduction of the intussusception, detorsion of any associated volvulus, and correction of malrotation through a Ladd’s procedure. When performed promptly, outcomes are excellent, even in cases involving extensive bowel involvement [4].

We present the case of a nine-year-old boy who initially presented with symptoms suggestive of viral gastroenteritis and was subsequently diagnosed with extensive ileo-colo-colic intussusception. Intraoperative findings revealed previously unrecognized intestinal malrotation consistent with Waugh syndrome. This case highlights the importance of maintaining a high index of suspicion in atypical presentations and underscores the role of early surgical exploration in preventing recurrence and complications.

## Case presentation

History and initial presentation

A nine-year-old boy from a rural area with no significant past medical history and no known drug allergies presented to the pediatric emergency department with a 24-hour history of diarrhea and vomiting, presumed to be due to an infectious gastroenteritis. He was clinically stable and well-hydrated; the pediatric team decided on symptomatic management at home. Laboratory tests at that time were within normal limits, and stool culture and ova/parasite examinations were negative.

Two days later, the patient returned due to persistent vomiting and new-onset diffuse, colicky abdominal pain. He was evaluated by the on-call pediatrician and admitted for further observation and intravenous fluid rehydration. During this admission, a viral stool panel tested positive for adenovirus and rotavirus (Table 1).

**Table 1:** Laboratory findings at admission

Parameter	Result	Reference Range
White blood cells	9.55 ×10³/µL	4.5-13.0 ×10³/µL
Neutrophils	84%	40-70%
Lymphocytes	12%	20-45%
Hemoglobin	14.3 g/dL	11.5-15.5 g/dL
Platelets	301 ×10³/µL	150-450 ×10³/µL
C-reactive protein	1.2 mg/L	<5 mg/L
Fibrinogen	384 mg/dL	200-400 mg/dL
Sodium	144 mEq/L	135-145 mEq/L
Potassium	4.1 mEq/L	3.5-5.0 mEq/L
Creatinine	0.70 mg/dL	0.4-0.9 mg/dL
Adenovirus (stool)	Positive	Negative
Rotavirus (stool)	Positive	Negative

Progressive symptoms and surgical evaluation

By day 5, the child’s symptoms had progressed: he continued to have intermittent vomiting, and he had passed no flatus for the past 12 hours. Given the worsening abdominal pain (still colicky, intensity 7/10) and abdominal distension, a pediatric surgical consultation was requested.

On examination by the surgical team, the patient appeared uncomfortable, lying in an antalgic position to relieve pain. He was adequately hydrated and alert but in moderate distress from pain. An abdominal exam revealed a palpable, tender mass in the central abdomen. There were no peritoneal signs. Digital rectal examination found an empty rectum.

**Figure 1:**
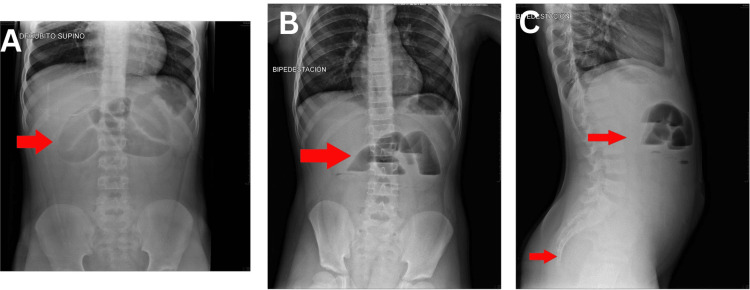
Abdominal X-ray A: supine view showing diffuse small-bowel distension (arrow); B: upright view demonstrating multiple air-fluid levels with paucity of distal gas (arrow); C: lateral upright view confirming air-fluid levels and absent rectal gas (arrows).

Initial imaging with plain abdominal X-rays (supine and upright films) showed multiple air-fluid levels in dilated loops of small intestine, with a paucity of gas in the colon and rectum, and no evidence of free air under the diaphragm (no pneumoperitoneum) (Figure 1). These findings suggested an intestinal obstruction. Given the patient’s age and these radiographic findings, an ileocolic intussusception was highly suspected as the cause of obstruction, possibly secondary to an underlying lead point.

Diagnostic imaging and findings

**Figure 2:**
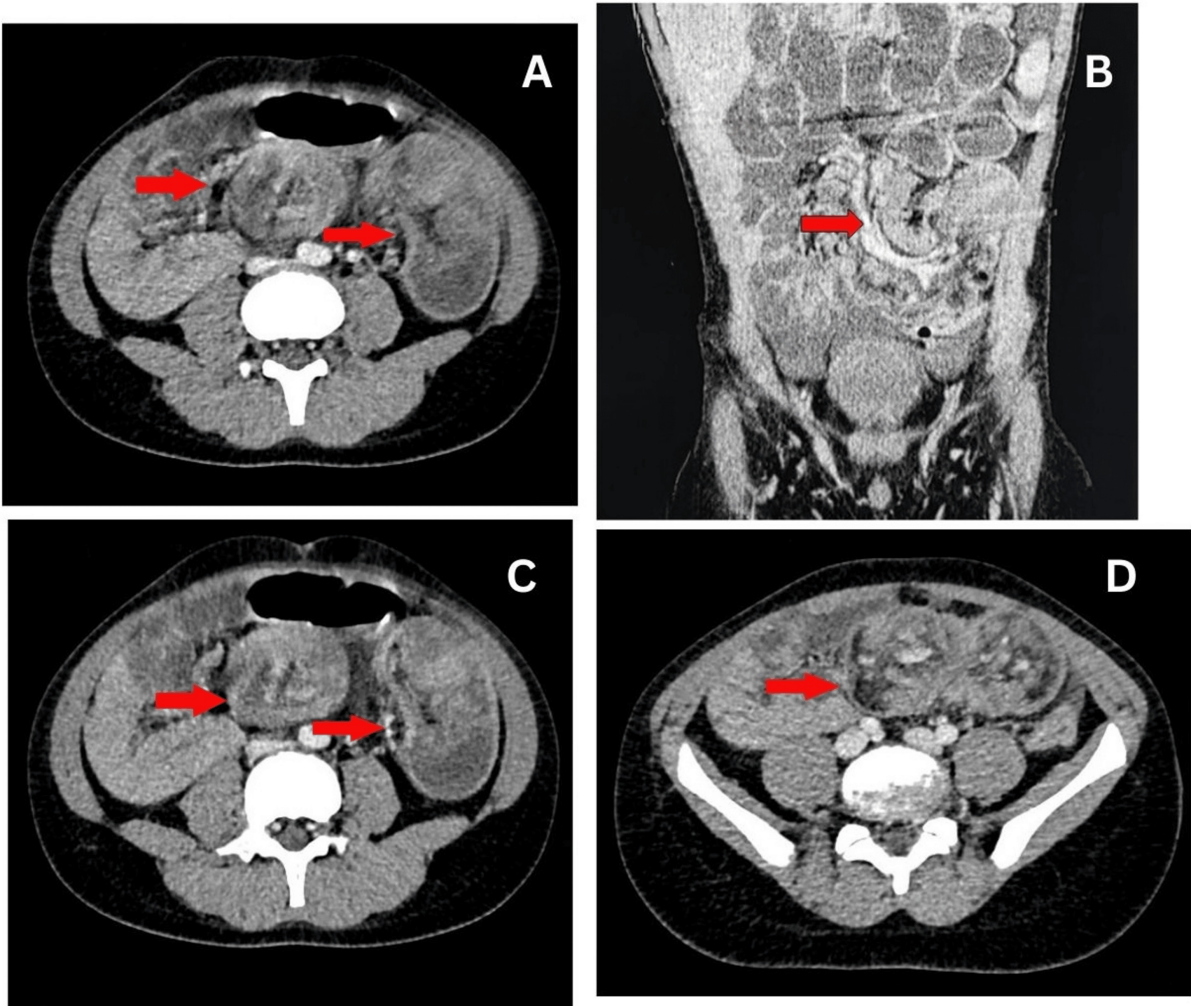
Computer tomography (CT) imaging A: axial image showing the “target sign” of intussusception with mesenteric lymphadenopathy (arrows); B: axial view demonstrating intussusception and a “whirlpool sign” consistent with midgut volvulus (arrow); C: axial image again showing the target sign (arrows); D: axial image highlighting enlarged mesenteric lymph nodes within the intussuscepted segment (arrow).

Further diagnostic workup was undertaken. Laboratory tests were repeated (including a metabolic panel and arterial blood gas analysis) to assess hydration status and electrolyte balance. The key investigation was a contrast-enhanced abdominal CT scan, which confirmed the presence of intussusception and also revealed anatomical features consistent with intestinal malrotation. Although the initial CT report did not mention any rotation anomaly, upon review, it was noted that the duodenum did not cross the midline as expected. The third portion of the duodenum (D3) was not in its normal retroperitoneal location; instead, it remained on the right side of the abdomen. These CT findings (Figure 2A-2D) indicated that the normal ligament of Treitz was absent or misplaced, confirming an underlying malrotation of the midgut. No signs of bowel ischemia were seen on imaging, and there was no evidence of bowel perforation.

Based on the combination of clinical and imaging findings, intussusception causing bowel obstruction in the setting of malrotation, the decision was made to proceed with prompt surgical exploration via a midline laparotomy.

Surgical findings and management

**Figure 3:**
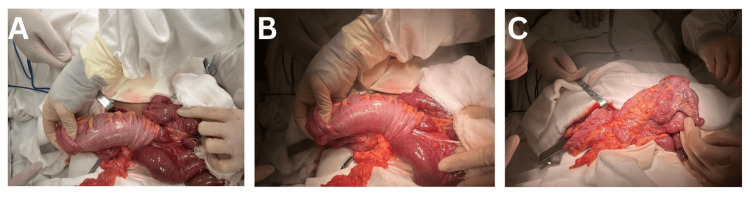
Intraoperative images A: ileocolic intussusception extending to the transverse colon with small-bowel congestion from 180° rotation; B: manual reduction after counterclockwise detorsion; C: reduced bowel with congested appendix and enlarged mesenteric lymph nodes.

A laparotomy was performed through a midline supra- and infra-umbilical incision (approximately 7 cm in length). Upon entering the peritoneal cavity, multiple abnormal findings were noted. An ileo-appendico-ceco-colic intussusception was present. In this large intussusception, the terminal ileum, appendix, cecum, and ascending colon had telescoped into the more distal colon. The intussuscepted segment extended to the distal third of the transverse colon (it had not reached the splenic flexure). Importantly, there were no signs of ischemia in the involved bowel. A midgut volvulus was also identified. The small bowel appeared to be twisted approximately 180° around the superior mesenteric artery axis. This had resulted in congestion and partial obstruction of the bowel, though fortunately, no vascular compromise was evident (the bowel remained perfused and viable) (Figure 3A). The overall orientation of the intestines confirmed an underlying intestinal malrotation. The small intestine was found predominantly on the right side of the abdomen, and the cecum was abnormally located in a subhepatic position. The normal duodenojejunal junction (ligament of Treitz) was not in its typical location (absent from the left upper quadrant). The ascending colon was mobile and not affixed to the posterior abdominal wall. Notably, there were no Ladd’s bands observed and no internal hernias present.

These findings explained the patient’s condition: the malrotation created a predisposition to midgut volvulus, and the concurrent viral illness with mesenteric lymphadenopathy likely served as a lead point triggering the ileocecal intussusception.

The surgical correction addressed all of these abnormalities. First, the midgut volvulus was detorsed by rotating the bowel counterclockwise, which immediately relieved the twist and improved bowel perfusion. Next, a gentle manual reduction of the intussusception was performed, successfully returning the telescoped segments to their normal anatomical position. During this reduction, the appendix, which had been drawn into the intussusception, was noted to be inflamed and swollen (erythematous, with a turgid tip). An appendectomy was therefore conducted to remove the compromised appendix (Figure 3B, 3C).

Finally, an elective Ladd’s procedure was carried out to correct the malrotation and prevent recurrence. The small bowel mesentery was widened, and the bowel anatomy was repositioned: the small intestines were placed on the right side of the abdomen, and the colon was placed on the left side. Since no obstructing Ladd’s bands were present, none needed to be divided. The abdominal cavity was inspected for any remaining abnormalities, and the incision was closed. The operation concluded without any intraoperative complications.

Postoperative course and outcome

The patient had an excellent recovery. He was started on oral fluids the day after surgery and advanced to a regular diet as tolerated. By 48 hours post-op, he was tolerating oral intake well and had passed stool, indicating a return of normal bowel function. He was discharged home two days after the operation in good condition.

In the follow-up evaluations (one week after and one month post-surgery), the patient was asymptomatic and had resumed normal activities. They continued to have regular bowel movements without abdominal pain or bloating. No late postoperative complications were observed.

This case illustrates a rare scenario of an older child with intussusception due to an underlying malrotation of the intestine. Timely diagnosis and surgical management (reduction of intussusception, detorsion of volvulus, and Ladd’s procedure) led to an excellent outcome for the patient.

## Discussion

Waugh syndrome, defined as the coexistence of intestinal malrotation and intussusception, is an exceptionally rare and likely underdiagnosed entity. Recent literature reviews estimate fewer than 100 reported cases worldwide, with an incidence under 1% among pediatric intussusceptions [1,2]. Its rarity contributes to diagnostic delay, particularly because many intussusceptions in infants are successfully reduced nonoperatively, potentially masking an underlying malrotation that remains unrecognized if surgical exploration is not performed [2,4]. Although intussusception classically occurs between three months and three years of age and is usually idiopathic, presentation outside this age range should prompt suspicion for a pathological lead point or anatomical anomaly [5]. In older children and adults, secondary causes are more frequently identified, and failure to investigate atypical presentations may result in incomplete treatment and recurrence [5,6]. Additionally, the episode occurred in the context of documented viral gastroenteritis (adenovirus and rotavirus). Viral infections, particularly adenovirus, are well-established triggers of intussusception due to lymphoid hyperplasia and mesenteric adenitis acting as transient lead points [3,7]. In a patient with malrotation, abnormal bowel mobility and a narrowed mesenteric base may further predispose to telescoping of the intestine [1]. This case, therefore, illustrates a convergence of factors: a viral inflammatory trigger superimposed on an anatomical predisposition, culminating in extensive intussusception [2,4].

Imaging reliably confirms intussusception, but associated malrotation is frequently overlooked if not specifically evaluated. Ultrasound and CT effectively identify the intussuscepted segment; however, radiologic signs of malrotation, such as failure of the duodenum to cross the midline or abnormal cecal positioning, may be subtle and omitted from reports. Several recent reviews emphasize that malrotation is often missed preoperatively because imaging interpretation focuses on the acute obstruction rather than bowel anatomy [1,4]. In our case, CT clearly demonstrated intussusception and mesenteric lymphadenopathy, yet malrotation was not reported until retrospective review revealed the abnormal duodenal course. These findings highlight the need for systematic evaluation of rotational anatomy in atypical or age-outlier presentations of intussusception [2,4].

The standard of care for a typical idiopathic ileocecal intussusception in an infant is nonoperative reduction under imaging guidance. This approach boasts high success rates and avoids surgery in most infants. Waugh syndrome, however, is not a typical intussusception scenario. When an intussusception is believed to be secondary to an anatomical anomaly or pathological lead point, many experts advocate for prompt surgical intervention rather than repeated nonoperative attempts [8].

Several recent case reports support this approach. Chukwubuike et al. described a seven-month-old infant with Waugh syndrome in whom hydrostatic reduction failed, necessitating laparotomy with manual reduction and correction of the underlying malrotation [9]. Similarly, Elkeir et al., in a case series of four pediatric patients, reported proceeding directly to operative management after diagnosis, performing manual reduction followed by a Ladd procedure in all cases, with excellent outcomes [10]. These reports reinforce the recommendation that atypical or secondary intussusceptions, particularly when associated with malrotation, should be managed with early surgical exploration rather than repeated nonoperative attempts.

In our nine-year-old patient, given his age, the large size and unusual type of intussusception, and CT findings suggestive of a possible lead point (enlarged lymph nodes), the team elected to proceed directly to surgical exploration. This decision aligns with literature recommendations that atypical intussusceptions warrant early operative management.

When Waugh syndrome is confirmed, simply reducing the intussusception is not sufficient, as the underlying malrotation must also be corrected to prevent future episodes and other complications. The gold-standard operation for intestinal malrotation is the Ladd procedure. A Ladd procedure is indicated whenever malrotation is present, even in older patients, because the risks of midgut volvulus or recurrent obstruction persist throughout life.

Dhungana et al. reported a four-year-old child initially misdiagnosed with gastroenteritis in whom Waugh syndrome was unexpectedly discovered intraoperatively; reduction of the intussusception alone was deemed insufficient, and a Ladd procedure was performed during the same operation [11]. Their report highlights both the potential for atypical presentations and the importance of addressing the malrotation component definitively at the time of surgery.

The key steps of the Ladd procedure include division of Ladd’s bands (peritoneal bands compressing the duodenum), widening of the mesenteric base to reduce the risk of volvulus, mobilization of the duodenum and right colon, and repositioning of the bowel, with the small intestine placed predominantly on the right side of the abdomen and the colon on the left. An appendectomy is routinely performed because, after bowel repositioning, the appendix lies in an atypical location; removing it prevents future diagnostic confusion in cases of abdominal pain.

In our patient, after manually reducing the ileoceco-colic intussusception, we performed an appendectomy and completed a Ladd’s procedure, leaving the small bowel predominantly on the right and the colon on the left. This intervention is curative: it addresses the malrotation that predisposed the intussusception, thereby dramatically reducing the risk of recurrence. This comprehensive approach addresses both the acute pathology and the underlying anatomical predisposition.

## Conclusions

Waugh syndrome remains a rare but clinically significant association between intussusception and intestinal malrotation. Presentation outside the typical age range for idiopathic intussusception should prompt thorough evaluation for secondary causes, including congenital rotational anomalies. Imaging studies may confirm intussusception but can overlook subtle signs of malrotation unless specifically assessed. Early recognition and comprehensive surgical management, including reduction of intussusception and correction of malrotation with a Ladd procedure, are essential to prevent recurrence and potentially life-threatening complications such as midgut volvulus. A systematic approach to atypical presentations can significantly improve diagnostic accuracy and patient outcomes.
